# Human Disease-Associated Genetic Variation Impacts Large Intergenic Non-Coding RNA Expression

**DOI:** 10.1371/journal.pgen.1003201

**Published:** 2013-01-17

**Authors:** Vinod Kumar, Harm-Jan Westra, Juha Karjalainen, Daria V. Zhernakova, Tõnu Esko, Barbara Hrdlickova, Rodrigo Almeida, Alexandra Zhernakova, Eva Reinmaa, Urmo Võsa, Marten H. Hofker, Rudolf S. N. Fehrmann, Jingyuan Fu, Sebo Withoff, Andres Metspalu, Lude Franke, Cisca Wijmenga

**Affiliations:** 1 Department of Genetics, University Medical Center Groningen, University of Groningen, Groningen, The Netherlands; 2 Institute of Molecular and Cell Biology and Estonian Genome Center, University of Tartu, Tartu, Estonia; 3 Graduate Program in Health Sciences, University of Brasilia School of Health Sciences, Brasilia, Brazil; 4 Molecular Genetics Section, Department of Pathology and Medical Biology, University Medical Center Groningen, University of Groningen, Groningen, The Netherlands; University of Pennsylvania, United States of America

## Abstract

Recently it has become clear that only a small percentage (7%) of disease-associated single nucleotide polymorphisms (SNPs) are located in protein-coding regions, while the remaining 93% are located in gene regulatory regions or in intergenic regions. Thus, the understanding of how genetic variations control the expression of non-coding RNAs (in a tissue-dependent manner) has far-reaching implications. We tested the association of SNPs with expression levels (eQTLs) of large intergenic non-coding RNAs (lincRNAs), using genome-wide gene expression and genotype data from five different tissues. We identified 112 *cis*-regulated lincRNAs, of which 45% could be replicated in an independent dataset. We observed that 75% of the SNPs affecting lincRNA expression (lincRNA *cis*-eQTLs) were specific to lincRNA alone and did not affect the expression of neighboring protein-coding genes. We show that this specific genotype-lincRNA expression correlation is tissue-dependent and that many of these lincRNA *cis*-eQTL SNPs are also associated with complex traits and diseases.

## Author Summary

Large intergenic non-coding RNAs (lincRNAs) are the largest class of non-coding RNA molecules in the human genome. Many genome-wide association studies (GWAS) have mapped disease-associated genetic variants (SNPs) to, or in, the vicinity of such lincRNA regions. However, it is not clear how these SNPs can affect the disease. We tested whether SNPs were also associated with the lincRNA expression levels in five different human primary tissues. We observed that there is a strong genotype-lincRNA expression correlation that is tissue-dependent. Many of the observed lincRNA *cis*-eQTLs are disease- or trait-associated SNPs. Our results suggest that lincRNA-eQTLs represent a novel link between non-coding SNPs and the expression of protein-coding genes, which can be exploited to understand the process of gene-regulation through lincRNAs in more detail.

## Introduction

It is now evident that most of the human genome is transcribed to produce not only protein-coding transcripts but also large numbers of non-coding RNAs (ncRNAs) of different size [1], [2]. Well-characterized short ncRNAs include microRNAs, small interfering RNAs, and piwi-interacting RNAs, whereas the large intergenic non-coding RNAs (lincRNAs) make up most of the long ncRNAs. LincRNAs are non-coding transcripts of more than 200 nucleotides long; they have an exon-intron-exon structure, similar to protein-coding genes, but do not encompass open-reading frames [3]. The recent description of more than 8,000 lincRNAs makes these the largest subclass of the non-coding transcriptome in humans [4].

Evidence is mounting that lincRNAs participate in a wide-range of biological processes such as regulation of epigenetic signatures and gene expression [5]–[7], maintenance of pluripotency and differentiation of embryonic stem cells [8]. In addition, several individual lincRNAs have also been implicated in human diseases. A well-known example is a region on chromosome 9p21 that encompasses an antisense lincRNA, ANRIL (antisense lincRNA of the *INK4* locus). Genome-wide association studies (GWAS) have shown that this region is significantly associated with susceptibility to type 2 diabetes, coronary disease, and intracranial aneurysm as well as different types of cancers [9] and some of the associated SNPs have been shown to alter the transcription and processing of ANRIL transcripts [10]. Similarly, increased expression of lincRNA HOTAIR (HOX antisense non-coding RNA) in breast cancer is associated with poor prognosis and tumor metastasis [10]. Another example is MALAT-1 (metastasis associated in lung adenocarcinoma transcript) where the expression is three-fold higher in metastasizing tumors of non-small-cell lung cancer than in non-metastasizing tumors [11].

In addition, over the last decade, more than 1,200 GWAS have identified nearly 6,500 disease- or trait-predisposing SNPs, but only 7% of these are located in protein-coding regions [12], [13]. The remaining 93% are located within non-coding regions [14], suggesting that GWAS-associated SNPs regulate gene transcription levels rather than altering the protein-coding sequence or protein structure. Even though there is growing evidence to implicate lincRNAs in human diseases [15], [16], it is unknown whether disease-associated SNPs could affect the expression of non-coding RNAs. We hypothesized that GWAS-associated SNPs can affect the expression of lincRNA genes, thereby proposing a novel disease mechanism.

To test this hypothesis, we performed eQTL mapping on 2,140 human lincRNA-probes using genome-wide gene expression and genotype data of 1,240 peripheral blood samples (discovery cohort) [17]. The lincRNA *cis*-eQTLs identified were then tested for replication in an independent cohort containing 891 peripheral blood samples (replication cohort). Since lincRNAs are considered to be more tissue-specific than protein-coding genes [4], we set-out to identify tissue-dependent *cis*-eQTLs for lincRNAs using data from another four different primary tissues from the subset of 85 individuals in our primary cohort [18]. Subsequently, we tested whether SNPs that affect the levels of lincRNA expression are associated with diseases or traits. Finally, we predicted the most likely function(s) of a subset of *cis*-eQTL lincRNAs by using co-regulation information from a compendium of approximately 80,000 expression arrays (www.GeneNetwork.nl).

## Results

### Commercial microarrays contain probes for a subset of non-coding RNA

Whole-genome gene expression oligonucleotide arrays have played a crucial role in our understanding of gene regulatory networks. Even though most of the currently available commercial microarrays are designed to capture all known protein-coding transcripts, they still include subsets of probes that capture transcripts of unknown function (sometimes abbreviated as TUF). We investigated whether the TUF probes present on the Illumina Human HT12v3 array, overlap with lincRNA transcripts that were recently described in the lincRNA catalog [4]. The lincRNA catalog contained a provisional set of 14,393 transcripts mapping to 8,273 lincRNA genes and a stringent set of 9,918 transcripts mapping to 4,283 lincRNA genes. We identified 2,140 unique probes that map to 1,771 different lincRNAs from the provisional set and 1,325 unique probes that map to 1,051 lincRNA genes from the stringent set. We chose 2,140 unique probes that mapped to lincRNAs from the provisional set for further eQTL analysis.

### Genetic control of lincRNAs expression in blood

It is known that in general lincRNAs are less abundantly expressed compared to protein-coding transcripts [4]. To test the expression levels of the 2,140 lincRNA probes in 1,240 peripheral blood samples (discovery cohort), we compared the quantile-normalized, log scale transformed mean expression intensity as well as expression variation of the lincRNA probes to probes mapping to protein-coding transcripts. We indeed observed a significant difference in the expression levels, where lincRNA probes are less abundant (mean expression = 6.67) than probes mapping to protein-coding transcripts (mean expression = 6.92, Wilcoxon Mann Whitney P<2.2×10^−16^; Figure S1). We also observed a highly significant difference in the expression variation between lincRNA probes and probes mapping to protein-coding transcripts (Wilcoxon Mann Whitney P<3.85×10^−96^). Next, we tested whether the expression of these 2,140 lincRNA probes is affected by SNPs in *cis*, by performing eQTL mapping in these 1,240 peripheral blood samples for which genotype data was also available. We confined our analysis to SNP-probe combinations for which the distance from the center of the probe to the genomic location of the SNP was ≤250 kb. In the end, at a false-discovery rate (FDR) of 0.05, we identified 5,201 significant SNP-probe combinations, reflecting 4,644 different SNPs; these affected the expression of 112 out of 2,140 different lincRNA probes. The 112 lincRNA probes mapped to 108 lincRNA genes and comprised 5.2% of all tested lincRNA probes, with a nominal significance ranging from *P*<2.8×10^−4^ to 9.81×10^−198^ in peripheral blood (Table S1).

### Replication of lincRNA *cis*-eQTLs in an independent blood dataset

We then performed a replication analysis to test the reproducibility of the identified 112 lincRNA *cis*-eQTLs using an independent dataset of 891 whole peripheral blood samples. We took the 112 lincRNA-probes (or 5,201 SNP-probe pairs) that were significantly affected by *cis*-eQTLs in the discovery cohort and tested whether these eQTLs were also significant in the replication dataset (at FDR 0.05). We could replicate 45% of the 112 lincRNA *cis*-eQTLs at an FDR<0.05, of which all the eQTLs had an identical allelic direction (Figure S2). The smaller sample size of the replication cohort compared to the discovery cohort makes it inherently difficult to replicate all the *cis*-eQTLs that we have detected in the discovery cohort.

### Number of *cis*-eQTLs is dependent on expression levels of transcripts

Our observation that 5.2% of all tested lincRNAs are *cis*-regulated (Table S1) might seem disappointing, compared to our earlier observation that 25% of the protein-coding probes in this dataset are *cis*-regulated [18]. However, we reasoned that the generally lower expression levels of lincRNAs compared to protein-coding genes might make it more difficult to detect *cis*-eQTLs for lincRNAs, as the influence of background noise becomes substantial for less abundant transcripts, making accurate expression quantification difficult (Figure S1A).

Indeed, we found significantly higher expression levels for the 112 *cis*-eQTL lincRNA probes (mean expression = 6.80) compared to the 2,028 non-eQTL lincRNA probes (mean expression = 6.66 Wilcoxon Mann Whitney P = 3.88×10^−15^; Figure S3) and also observed a significant difference in expression variance between the 112 *cis*-eQTL lincRNAs compared to the 2,028 non-*cis* eQTL lincRNAs (Wilcoxon Mann Whitney P = 1.067×10^−8^), indicating that lower overall expression levels do make identification of *cis*-eQTLs more difficult.

**Figure 1:**
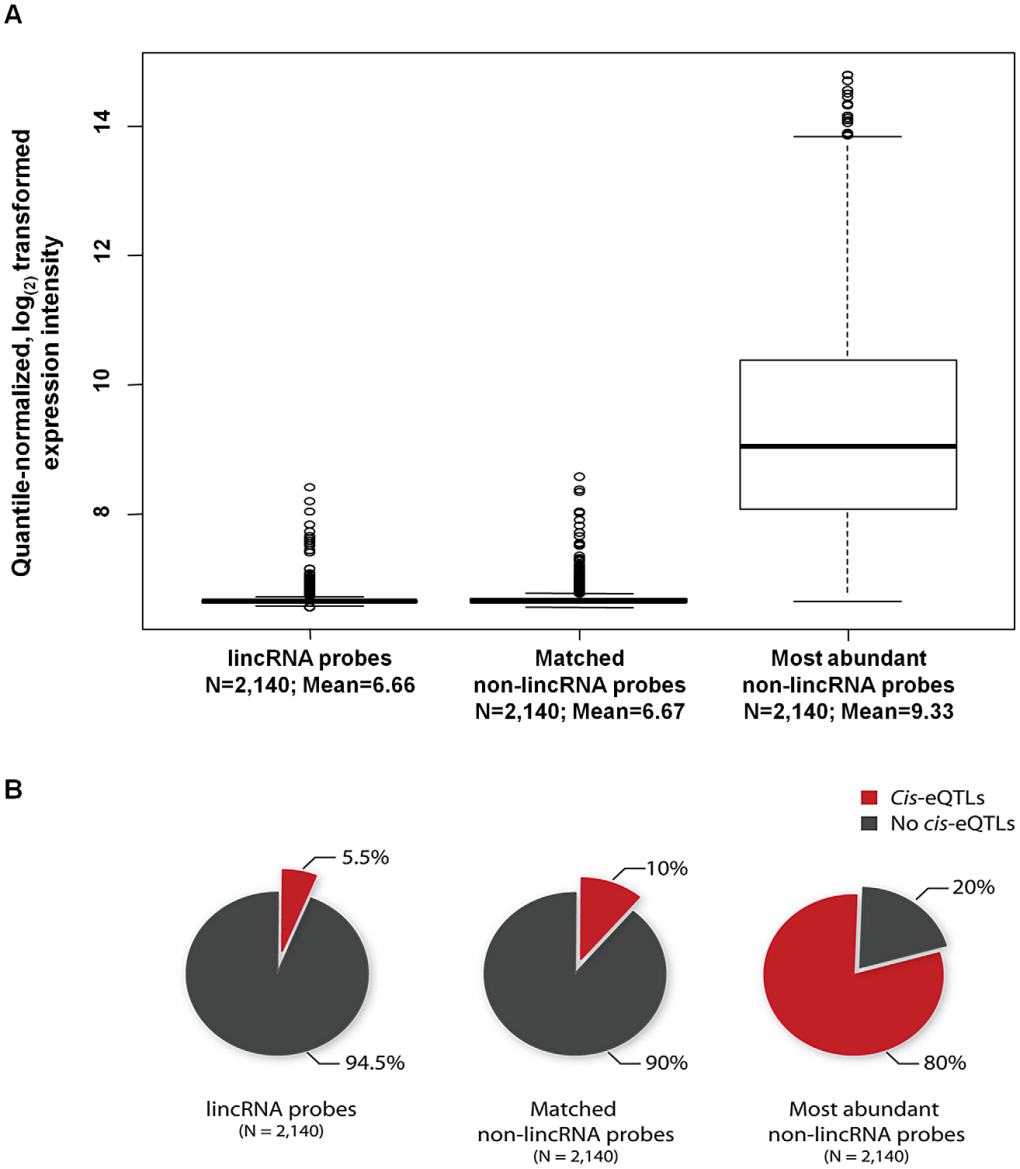
The number of detected *cis*-eQTLs is dependent on the expression levels of the transcripts. (A) Quantile-normalized average expression intensity and (B) number of *cis*-eQTL affected probes in percentage, for 2,140 lincRNA probes, 2,140 non-lincRNA (matched for 2,140 lincRNA probes' median expression and standard deviation) and 2,140 most abundantly expressed non-lincRNA probes.

To further confirm the relationship between average expression levels of probes and the number of detectable *cis*-eQTLs, we first mapped *cis*-eQTLs for an equal set of 2,140 probes that were instead protein-coding and were the most abundantly expressed of all protein-coding probes. We also conducted *cis*-eQTL mapping for a set of 2,140 protein-coding probes that had been selected to have an identical expression intensity distribution as the 2,140 lincRNA probes (i.e. matched for mean expression intensity and standard deviation), using the same 1,240 blood samples (Figure 1A). We indeed observed a profound relationship between average expression levels of protein-coding transcripts and the number of detectable *cis*-eQTLs. Eighty percent of the 2,140 most abundantly expressed protein-coding probes showed a *cis*-eQTL effect, whereas only 10% of the protein-coding probes that had been matched for an expression intensity of the 2,140 lincRNA-probes were affected by *cis*-eQTLs (Figure 1B).

Hence it is possible that if we can accurately quantify all lincRNAs in large RNA-sequencing datasets, we will be able to identify *cis*-eQTLs for a larger proportion of all lincRNAs.

### Most SNPs that affect lincRNA expression do not alter the expression of protein-coding genes

It could be possible that the SNPs that affect lincRNA expression actually operate by first affecting protein-coding gene expression levels, which in turn affect lincRNA expression. If this were to be the case, our identified lincRNA *cis*-eQTLs would merely be a by-product of protein-coding *cis*-eQTLs. To ascertain this, we tested whether the 112 lincRNA-eQTL SNPs were also significantly affecting neighboring protein-coding genes. By keeping the same significance threshold (at FDR<0.05 level, the *P*-value threshold was 2.4×10^−4^), we observed that nearly 75% (83 out of 112) of the lincRNA-eQTLs were affecting only lincRNAs, even though the interrogated neighboring protein-coding genes were generally more abundantly expressed than the lincRNAs themselves (Figure S4). Genetic variants can thus directly regulate the expression levels of lincRNAs.

We found 29 *cis*-eQTLs to be associated with the expression of both lincRNA and protein coding genes. For 50% of these 29 *cis*-eQTLs, we found that the expression of lincRNAs and protein-coding genes was in the opposite direction, whereas for the other 50% of *cis*-eQTLs, both types of transcripts were co-regulated in the same direction (Figure S5).We tested whether these 29 *cis*-eQTLs are the strongest eQTLs for both lincRNA and protein-coding genes. Although these 29 *cis*-eQTLs were the strongest eQTLs for lincRNAs, only 5 among 29 were also the strongest eQTLs for protein-coding genes. This observation further highlights the direct regulation of lincRNA expression through genetic variants.

### Some lincRNA *cis*-eQTLs are tissue-dependent

There is considerable interest in mapping eQTLs in disease-relevant tissue types. We reasoned that since expression of the lincRNAs seems to be much more tissue-specific than the expression of protein-coding genes [4], mapping lincRNA-eQTLs in different tissues could reveal additional, tissue-specific lincRNA-eQTLs. To test this, we analyzed gene expression and genotype data of 74 liver samples, 62 muscle samples, 83 subcutaneous adipose tissue (SAT) samples, and 77 visceral adipose tissue (VAT) samples from our primary cohort of 85 unrelated, obese Dutch individuals [18]. Upon *cis*-eQTL mapping we detected 35 *cis*-eQTL-probes, of which 18 were specific in the four different non-blood tissues, resulting in a total of 130 lincRNA-eQTLs in the combined set of all five tissues (Table S1). Five *cis*-eQTLs identified in blood tissue were also significantly replicated in at least one other non-blood tissue (Table S1). While we could replicate 45% of the *cis*-eQTLs in the substantial whole peripheral blood replication cohort, the replication rate in the very small cohorts for fat, liver and muscle tissue was, as expected, much lower. We were able to observe tissue-specific lincRNA eQTLs in muscle (1), liver (4), SAT (9) and blood (107) (Figure S6). Since the four non-blood tissue expression levels were from the same individuals, these results do indeed provide evidence that some of the lincRNAs are regulated by genetic variants in a tissue-specific manner.

### LincRNA tissue specific *cis*-eQTLs are disease-associated SNPs

As most of the GWAS-associated SNPs are located within non-coding regions, we tested whether the 130 lincRNA-eQTLs identified in five different tissues are also GWAS-associated variants. To do this, we intersected trait-associated SNPs (at reported nominal *P*<9.9×10^−6^, retrieved from the catalog of published genome-wide association studies per 26 July 2012) [14] with the 130 top lincRNA *cis*-eQTLs and their proxies (proxies with R^2^>0.8 using the 1000Genome CEU population as reference). We identified 12 GWAS SNPs or their proxies, that were also a lincRNA *cis*-eQTLs of eight different lincRNA genes (Table 1). All except one of the 12 SNPs were exclusively associated with lincRNA expression and thus did not affect the expression levels of neighboring protein-coding genes (Table 1), suggesting a causative role of altered lincRNA expression for these phenotypes.

**Table 1:** Some of the lincRNA *cis*-eQTLs are disease-associated SNPs.

*Cis*-eQTL SNP	eQTL *P* on lincRNA	Proxies (R^2^>0.8) associated with disease/trait	Chr	Trait/Disease	eQTL affected lincRNA	eQTL tissue
rs13278062	4.31×10^−32^	rs13278062	8	Exudative age-related macular degeneration	XLOC_006742	Blood
rs11066054	4.09×10^−11^	rs6490294	12	Mean platelet volume	XLOC_010202	Blood
rs206942	3.63×10^−5^	rs206936	6	Body mass index	XLOC_005690	Blood
rs11065766	6.67×10^−5^	rs10849915	12	Alcohol consumption	XLOC_009878	Blood
	6.67×10^−5^	rs10774610	2	Drinking behavior		
rs1465541	1.84×10^−4^	rs11684202	2	Coronary heart disease	XLOC_002026	Blood
rs12125055	1.84×10^−4^	rs7542900	1	Type 2 diabetes	XLOC_000922	Blood
rs199439	8.25×10^−6^	rs199515	17	Parkinson's disease	XLOC_012496	SAT
		rs415430	17	Parkinson's disease		SAT
		rs199533	17	Parkinson's disease		SAT
rs17767419	1.05×10^−8^	rs17767419	16	Thyroid volume	XLOC_011797	SAT, VAT
		rs3813582	16	Thyroid function		SAT, VAT

*Chr chromosome, SAT Saturated adipose tissue, VAT Visceral adipose tissue*.

Notably SNP rs13278062 at 8p21.1, associated with exudative age-related macular degeneration (AMD) in the Japanese population, was reported to alter the transcriptional levels of *TNFRSF10A* (Tumor necrosis factor receptor superfamily 10A) protein-coding gene [19]. Here we identified SNP rs13278062 as a highly significant *cis*-eQTL of lincRNA XLOC_006742 (LOC389641) (P = 4.31×10^−32^) rather than for *TNFRSF10A* (P = 4.21×10^−4^) protein-coding gene (Figure S7). Furthermore, SNP rs13278062 is located in exon 1 of lincRNA XLOC_006742, which encompasses an ENCODE (Encyclopedia of DNA elements) enhancer region characterized by H3K27acetylation and DNaseI hypersensitive clusters [20] (Figure S8).

Another interesting example is at 17q21.31 where three Parkinson's disease associated SNPs were in strong linkage disequilibrium (R^2^>0.8) with top *cis*-eQTL SNP rs199439, which affects lincRNA XLOC_012496 expression exclusively in SAT (Table 1). Weight loss due to body-fat wasting is a very common but poorly understood phenomenon in Parkinson's disease patients [21]. In this regard, it is intriguing to note that the Parkinson's disease associated SNPs affects lincRNA expression exclusively in fat tissue (Table 1). Hence, identifying lincRNA-eQTLs in disease-relevant tissue types using larger groups of individuals may open up new avenues towards achieving a better understanding of disease mechanisms.

### LincRNA function predictions using a co-expression network of ∼80,000 arrays: A mechanistic link between disease and lincRNA

Our observations suggest a role for lincRNAs in complex diseases and other phenotypes. The next, rather daunting task is to elucidate the function of these ncRNAs. We recently developed a co-regulation network (GeneNetwork, www.genenetwork.nl/genenetwork, *manuscript in preparation*), to predict the function of any transcript based on co-expression data extracted from approximately 80,000 Affymetrix microarray experiments (see Methods). We interrogated the GeneNetwork database to predict the function of eQTL-affected lincRNAs. Among the 130 *cis*-eQTL lincRNAs that we had identified in the five different tissues, 43 were represented by expression probe sets on Affymetrix arrays for which we could predict the function (Table S2). These 43 probes include four out of eight disease-associated lincRNAs described above (Table 1) and function prediction for these probes provided relevant biological explanations.

### LincRNA co-expression analysis: Disease-associated lincRNAs are co-expressed with neighboring protein-coding genes

It has been reported that some transcribed long ncRNAs function as enhancers that regulate the expression of neighboring genes [3] and may thereby contribute to the disease pathology. We found that the AMD-associated lincRNA XLOC_006742 (LOC389641) (by virtue of SNP rs13278062 which exhibits a significant eQTL effect) (Figure S7) is in strong co-expression with *TNFRSF10A* based on our GeneNetwork database (Table S3). AMD is a leading cause of blindness among elderly individuals worldwide and recent studies, both in animal models and in humans, provide compelling evidence for the role of immune system cells in its pathogenesis [22]. The gene *TNFRSF10A*, which encodes TRAIL receptor 1 (TRAIL1), has been implicated as a causative gene for AMD [19]. It has been shown that binding of TRAIL to TRAILR1 can induce apoptosis through caspase 8 activation [23] and using GeneNetwork we also predict a role in apoptosis for lincRNA XLOC_006742 (Table S2).

Another trait-associated SNP, rs11065766, is the top *cis*-eQTL of lincRNA XLOC_009878 (ENSG00000185847 or RP1-46F2.2 or LOC100131138) and it is in strong linkage disequilibrium with two SNPs associated with alcohol drinking behavior (Table 1). We found that the lincRNA XLOC_009878 is strongly co-expressed with the neighboring protein-coding gene *MYL2* (Table S4) and, according to our predictions, lincRNA XLOC_009878 is involved in striated muscle contraction (P = 1.22×10^−26^). Chronic alcohol abuse can lead to striking changes in skeletal muscle structure, which in turn plays a role in the development of alcoholic myopathy and/or cardiomyopathy [24]. It has also been reported that alcohol can reduce the content of skeletal muscle proteins such as titin and nebulin to affect muscle function in rats [25]. We found lincRNA XLOC_009878 to be co-expressed with titin and many other skeletal muscle proteins necessary for the structural integrity of the muscle (Table S4). Thus, it needs to be tested whether deregulation of lincRNA XLOC_009878 expression might alter an individual's ability to metabolize alcohol due to changes in the muscle functional property.

### Localization of lincRNA *cis*-eQTLs in regulatory regions

**Figure 2:**
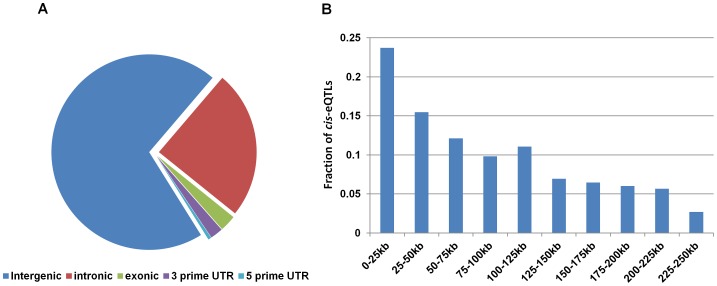
Distribution of lincRNA *cis*-eQTLs with respect to different transcripts. (A) The majority of the lincRNA *cis*-eQTLs are located within the non-coding part of the genome and less than 6% of lincRNA *cis*-eQTLs are located within mRNA. (B) Distribution of lincRNA *cis*-eQTLs with respect to distance to the lincRNA transcripts. The x-axis displays the 250 kb window used for *cis*-eQTL mapping and the y-axis displays the fraction of lincRNA *cis*-eQTLs located within this window.

**Figure 3:**
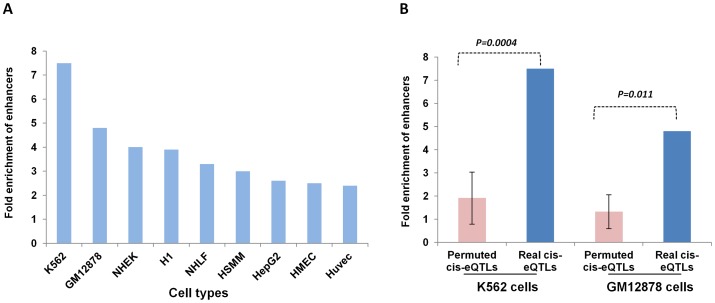
Localization of lincRNA *cis*-eQTLs in regulatory regions. (A) A plot to indicate the location of lincRNA *cis*-eQTLs in cell-specific enhancers. The x-axis shows the different cell lines analyzed and the y-axis shows the fold enrichment of enhancers. (B) A plot to show the difference in fold enrichment of enhancers for real lincRNA *cis*-eQTLs compared to permuted lincRNA *cis*-eQTLs. The significance of the difference in fold enrichment was tested by T-test. The HaploReg database was used to analyze the fold enrichment of enhancers.

We found that more than 70% of the lincRNA *cis*-eQTLs from both blood and non-blood tissues were located in intergenic regions with respect to protein-coding genes (Figure 2A). We also found high frequencies of lincRNA *cis*-eQTLs to be located around transcriptional start site (Figure 2B), suggesting that these *cis*-eQTLs may affect the expression of lincRNAs through similar gene regulatory mechanisms as those seen for protein-coding *cis*-eQTLs. Thus, in order to understand the mechanism of how lincRNA *cis*-eQTLs affect lincRNA expression, we intersected the location of top 112 lincRNA *cis*-eQTLs and their proxies (r^2^ = 1) in blood with regulatory regions using the HaploReg database [26]. The results suggested that indeed most of the lincRNA *cis*-eQTLs (69%) were located in functionally important regulatory regions (Figure S8), which contained DNAse I regions, transcription factor binding regions, and histone marks of promoter and enhancer regions. Furthermore, these *cis*-eQTLs were found to be located more often within blood cell-specific enhancers (K562 and GM12878) (Figure 3A), suggesting that some of these *cis*-eQTLs regulate lincRNA expression in a tissue-specific manner through altering these enhancer sequences.

Since we observed enrichment of cell-specific enhancers for lincRNA *cis*-eQTLs within blood cells (K562 and GM12878), we compared the fold enrichment of enhancers in these two cell types to see whether lincRNA *cis*-eQTLs are more often located in functionally important regions than any random set of SNPs. We found a significant difference in the enrichment of enhancers in which more than a 4-fold enrichment was seen for real *cis*-eQTLs both in K562 cells (P = 0.0004) and GM12878 cells (P = 0.011) compared to permuted SNPs. These findings suggest that some of the identified lincRNA *cis*-eQTLs are indeed functional SNPs.

## Discussion

Even though it may have been expected that lincRNA expression would be under genetic control, this is the first study, to our knowledge, to comprehensively establish this link. We were able to identify *cis*-eQTLs in five different tissues and have demonstrated that common genetic variants regulate the expression of lincRNAs alone. It is intriguing that around 75% of lincRNA *cis*-eQTLs are specific to lincRNAs alone, but not to protein-coding genes. Recent data from the ENCODE project suggests that combinations of different transcription factors are involved in regulating gene-expression in different cell types and non-coding RNAs tend to be regulated by certain combinations of transcription factors more often than others [27]. Thus, it could still be possible that some transcription factors specifically regulate lincRNA expression. We also observed a strong relationship between whether or not a transcript is affected by *cis*-eQTLs and its expression levels, where highly abundant transcripts were more often affected by *cis*-eQTLs. This relationship was comparable between lincRNA and protein-coding probes, although protein-coding probes (matched for expression levels of lincRNA probes) tend to show more *cis*-eQTLs (Figure 1B; 5.2% versus 10%). Although this difference is not drastic, it may suggest that lincRNAs exhibit another layer of gene regulation which is more tissue-specific. Thus, we may expect to identify many more lincRNA *cis*-eQTLs once larger datasets of different tissues become available.

One limitation of our study is the lack of probes to comprehensively map eQTLs to all the reported lincRNAs, as we relied upon microarrays. Future analyses using RNA-sequencing datasets will undoubtedly provide much more insight into how genetic variants affect lincRNA expression. So far, two landmark RNA-sequencing based eQTL studies have been published using 60 (Montgomery et al) [28] and 69 samples (Pickrell et al) [29], respectively. While Pickrell et al did not mention lincRNAs with a *cis*-eQTL effect, Montgomery et al identified six *cis*-regulated lincRNAs (at a slightly higher FDR of 0.17). We re-analyzed these two datasets and found that we could replicate one of the 112 *cis*-eQTL lincRNAs effects that we detected using arrays (with an identical allelic direction; Figure S10). These results indicate that *cis*-eQTL lincRNAs detected using conventional microarrays can be replicated in sequencing-based datasets. However, it also indicates that sample size is currently a limiting factor in finding many more *cis*-eQTL lincRNAs in sequencing-based datasets.

Nevertheless, our results clearly indicate that there is a strong genotype-lincRNA expression correlation that is tissue-dependent. A considerable number of the observed lincRNA *cis*-eQTLs are disease- or trait-associated SNPs. Since lincRNAs can regulate the expression of protein-coding genes either in *cis* [3] or in *trans* [8], lincRNA-eQTLs represent a novel link between non-coding SNPs and the expression of protein-coding genes. Our examples show that this link can be exploited to understand the process of gene-regulation in more detail, which may assist us in characterizing lincRNAs as another class of disease biomarkers.

## Methods

### Ethics statement

This study was approved by the Medical Ethical Board of Maastricht University Medical Center (four non-blood tissues), and local ethical review boards (1,240 peripheral blood samples) in line with the guidelines of the 1975 Declaration of Helsinki. Informed consent in writing was obtained from each subject personally. The subject information is provided in Table S5.

### Mapping probes to lincRNAs

A detailed mapping strategy of Illumina expression probe sequences has been described previously [17]. We extracted 43,202 expression probes mapping to single genomic locations (hg18 build) and excluded those that did not map or that mapped to multiple different loci. LincRNA chromosomal coordinates (hg19 build) were obtained from the lincRNA catalog (http://www.broadinstitute.org/genome_bio/human_lincrnas/?q=lincRNA_catalog) and converted to hg18 coordinates using UCSC's LiftOver application (http://genome.ucsc.edu/cgi-bin/hgLiftOver). Subsequently, we extracted probes mapping to lincRNA exonic regions by employing BEDtools [30].

### Blood dataset of 1,240 samples

The blood dataset and a detailed eQTL mapping strategy have been described previously [17]. Briefly, 1,240 peripheral blood samples from unrelated, Dutch control subjects were investigated (Table S5). Genotyping of these samples was performed according to Illumina's standard protocols (Illumina, San Diego, USA), using either the HumanHap370 or 610-Quad platforms. Because the non-blood samples (see below) were genotyped using Illumina HumanOmni1-Quad BeadChips, we applied IMPUTE v2 [31] to impute the genotypes of SNPs that were covered by the Omni1-Quad chip but that were not included on the Hap370 or 610-Quad platforms [31]. Anti-sense RNA was synthesized using the Ambion Illumina TotalPrep Amplification Kit (Ambion, New York, USA) following the manufacturer's protocol. Genome-wide gene expression data was obtained by hybridizing complementary RNA to Illumina's HumanHT-12v3 array and subsequently scanning these chips on the Illumina BeadArray Reader.

### Replication blood dataset of 891 samples

We used a dataset comprising peripheral blood samples of 891 unrelated individuals from the Estonian Genome Centre, University of Tartu (EGCUT) biobank cohort of 53,000 samples for replication. Genotyping of these samples was performed according to Illumina's standard protocols, using Illumina Human370CNV arrays (Illumina Inc., San Diego, US), and imputed using IMPUTE v2 [31], using the HapMap CEU phase 2 genotypes (release #24, build 36). Whole peripheral blood RNA samples were collected using Tempus Blood RNA Tubes (Life Technologies, NY, USA), and RNA was extracted using Tempus Spin RNA Isolation Kit (Life Technologies, NY, USA). Quality was measured by NanoDrop 1000 Spectrophotometer (Thermo Fisher Scientific, DE, USA) and Agilent 2100 Bioanalyzer (Agilent Technologies, CA, USA). Whole-Genome gene-expression levels were obtained by Illumina Human HT12v3 arrays (Illumina Inc, San Diego, US) according to manufacturers' protocols.

### Four non-blood primary tissues

Previously we described tissue-dependent eQTLs in 74 liver samples, 62 muscle samples, 83 SAT samples and 77 VAT samples from a cohort of 85 unrelated, obese Dutch individuals (all four tissues were available for 48 individuals) [18] (Table S5). These samples were genotyped according to standard protocols from Illumina, using Illumina HumanOmni-Quad BeadChips (Omni1). Genome-wide gene expression data of all samples was assayed by hybridizing complementary RNA to the Illumina HumanHT-12v3 array and then scanning it on the BeadArray Reader.

### *Cis*-eQTL mapping

The method for normalization and principal component analysis-based correction of expression data, along with the methods to control population stratification and SNP quality, were described previously [17], [18]. The *cis*-eQTL analysis was performed on probe-SNP combinations for which the distance from the center of the probe to the genomic location of the SNP was ≤250 kb. Associations were tested by non-parametric Spearman's rank correlation test and the *P* values were corrected for multiple testing by false-discovery rate (FDR) at *P*<0.05, in which the distribution was obtained from permuting expression phenotypes relative to genotypes 100 times within the HT12v3 dataset and comparing those with the observed *P*-value distribution. At FDR = 0.05 level, the *P*-value threshold was 2.4×10^−4^ for significantly associated probe-SNP pairs in blood, 1.5×10^−5^ in SAT, 5.21×10^−6^ in VAT, 6.3×10^−6^ in liver and 1.8×10^−6^ in muscle.

### LincRNA function prediction

To predict the function(s) for lincRNAs, we interrogated the GeneNetwork database (www.genenetwork.nl/genenetwork) that has been developed in our lab (*manuscript in preparation*). In short, this database contains data extracted from approximately 80,000 microarray experiments that is publically available from the Gene Expression Omnibus; after extensive quality control, it contains data on 54,736 human, 17,081 mouse and 6,023 rat Affymetrix array experiments. Principal component analysis was performed on probe-set correlation matrices of each of four platforms (two human platforms, one mouse and one rat platform), resulting in 777, 377, 677 and 375 robust principal components, respectively. Jointly these components explain between 79% and 90% of the variance in the data, depending on the species or platform. Many of these components are well conserved across species and enriched for known biological phenomena. Because of this, we were able to combine the results into a multi-species gene network with 19,997 unique human genes, allowing us to utilize the principal components to accurately predict gene function by using a ‘guilt-by-association’ procedure (a description of the method is available at www.genenetwork.nl/genenetwork). Predictions were made based on pathways and gene sets from Gene Ontology, KEGG, BioCarta, TransFac and Reactome.

### Functional annotation of lincRNA *cis*-eQTLs

We employed the HaploReg web tool [26] to intersect SNPs (and their perfect proxies, r^2^ = 1 using the CEU samples from the 1000 Genomes project) with regulatory information and also to calculate the fold enrichment of cell-type specific enhancers. In order to ascertain whether this enrichment was higher than expected, we took eQTL results from 100 permutations (shuffling the gene expression identifier labels): for each permutation we determined the top 112 eQTL probes and took the corresponding top SNPs and their perfect proxies (r^2^ = 1). We extracted the fold enrichment of enhancers from HaploReg for these 100 sets of SNPs as well, which then permitted us to estimate the significance of enrichment of the real eQTL analysis, determined by fitting a normal distribution on the 100 log-transformed permutation enrichment scores.

## Supporting Information

Figure S1 LincRNA probes show different expression characteristics compared to other transcripts. The figure shows the difference in quantile-normalized average expression intensity between lincRNA probes and non-lincRNA probes. The significance of difference in expression intensity was tested by the Wilcoxon Mann Whitney test.
(TIF)

Figure S2 Replicated lincRNA cis-eQTLs show identical allelic direction of effect in the both the discovery and replication datasets. We compared the z-scores (association strength) of each significantly associated probe-SNP pair in the discovery dataset (Groningen HT12v3; N = 1,240) with the replication dataset (EGCUT; N = 891).
(TIF)

Figure S3 lincRNA probes with *cis*-eQTL effect show higher expression levels compared to lincRNA probes without *cis*-eQTL effect. The significance of difference in expression intensity was tested by the Wilcoxon Mann Whitney test.
(TIF)

Figure S4 LincRNA *cis*-eQTL SNPs mostly affect lincRNA transcripts alone. Quantile-normalized average expression intensity of *cis*-eQTL lincRNAs and their neighboring protein coding genes without *cis*-eQTL.
(TIF)

Figure S5 Distribution of Z-scores of co-regulated lincRNA and protein-coding genes. We compared the z-scores (association strength) of each significantly associated probe-SNP pair for the 29 *cis*-eQTLs that affect both lincRNAs and protein-coding genes.
(TIF)

Figure S6 Number of specific and overlapping *cis*-eQTL lincRNAs identified across five different tissues.
(TIF)

Figure S7 Plots to show the association of age-related macular degeneration SNP rs13278062 with expression levels of lincRNA LOC389641 and protein-coding gene *TNFRSF10A* in blood (N = 1,249). The x-axis shows the number of samples according to the genotypes at rs13278062 and the y-axis is the average expression intensity of probes.
(TIF)

Figure S8 UCSC genome browser screen shot (http://genome.ucsc.edu) to show the location of age-related macular degeneration SNP, rs13278062. The x-axis is the chromosome location in the hg19 build and indicates the location of transcripts and regulatory elements identified by ENCODE on chromosome 8.
(TIF)

Figure S9 A plot to show the number of lincRNA *cis*-eQTLs on the y-axis within different regulatory regions on the x-axis.
(TIF)

Figure S10 Plots to show the *cis*-eQTL effect on lincRNA XLOC_00197 from both microarray data (Groningen HT12v3; N = 1,240) and RNA-sequencing data (Montgomery et al; N = 60). The x-axis shows the number of samples according to the genotypes at rs1120042 and rs2279692 (LD between these two SNPs, R^2^ = 0.96) in microarray data and RNA-sequencing data, respectively.
(TIF)

Table S1 LincRNA *cis*-eQTLs in blood and four other non-blood tissues.
(XLSX)

Table S2 Function prediction of lincRNAs affected by *cis*-eQTLs using GeneNetwork.
(XLSX)

Table S3 Identification of co-expressed genes for lincRNA LOC389641 using GeneNetwork.
(XLSX)

Table S4 Identification of co-expressed genes for lincRNA LOC100131138 using GeneNetwork.
(XLSX)

Table S5 Characteristics of sample cohorts used for *cis*-eQTL mapping.
(XLSX)
