# Supplementary figures and images for: Human Disease-Associated Genetic Variation Impacts Large Intergenic Non-Coding RNA Expression
Source: PLoS Genet. 2013 Jan 17;9(1):e1003201. doi: 10.1371/journal.pgen.1003201 (PMC3547830; doi:10.1371/journal.pgen.1003201)

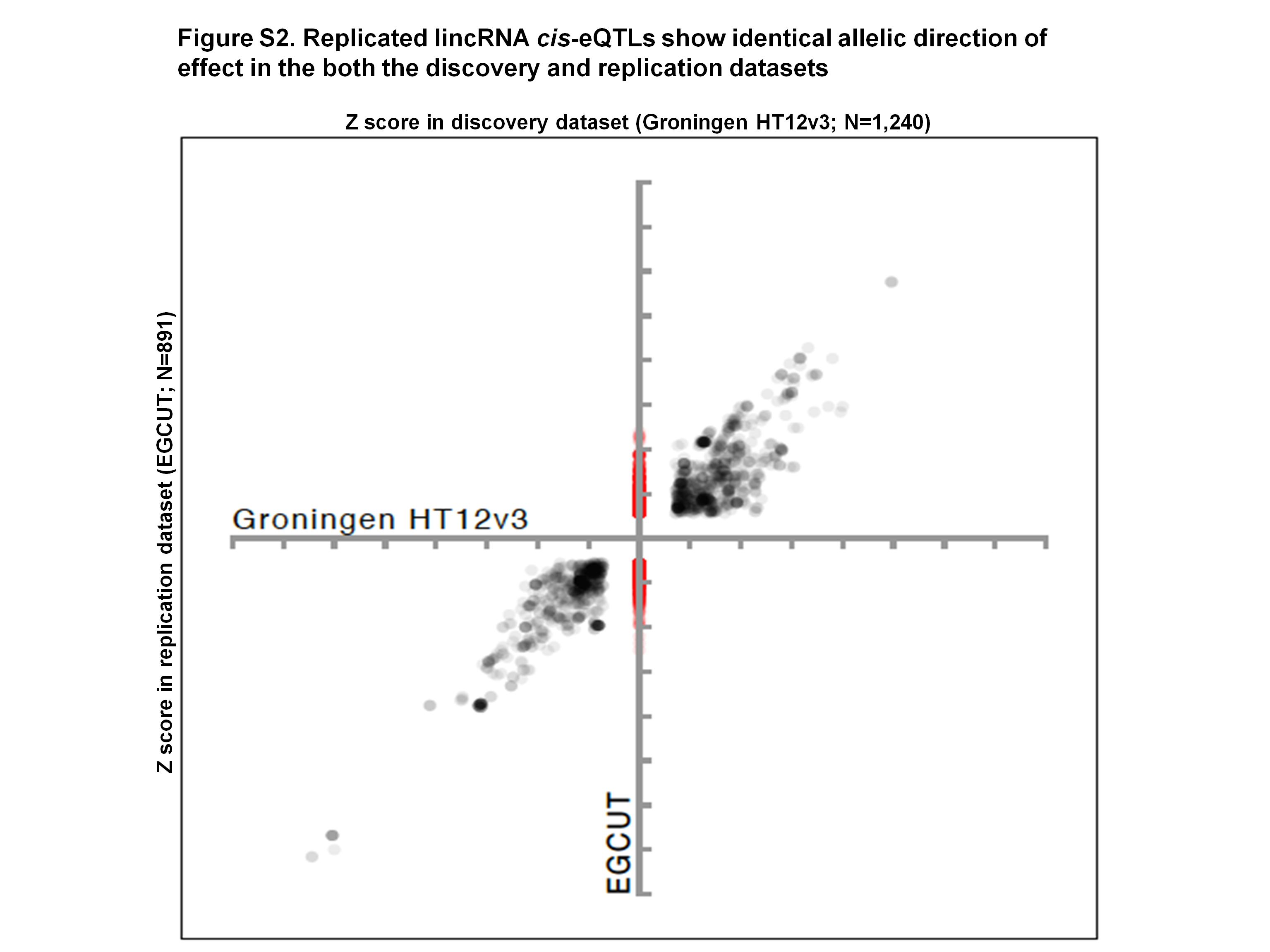

Supplement: Figure S2 — Replicated lincRNA cis-eQTLs show identical allelic direction of effect in the both the discovery and replication datasets. We compared the z-scores (association strength) of each significantly associated probe-SNP pair in the discovery dataset (Groningen HT12v3; N = 1,240) with the replication dataset (EGCUT; N = 891). (TIF) [file pgen.1003201.s002.tif]

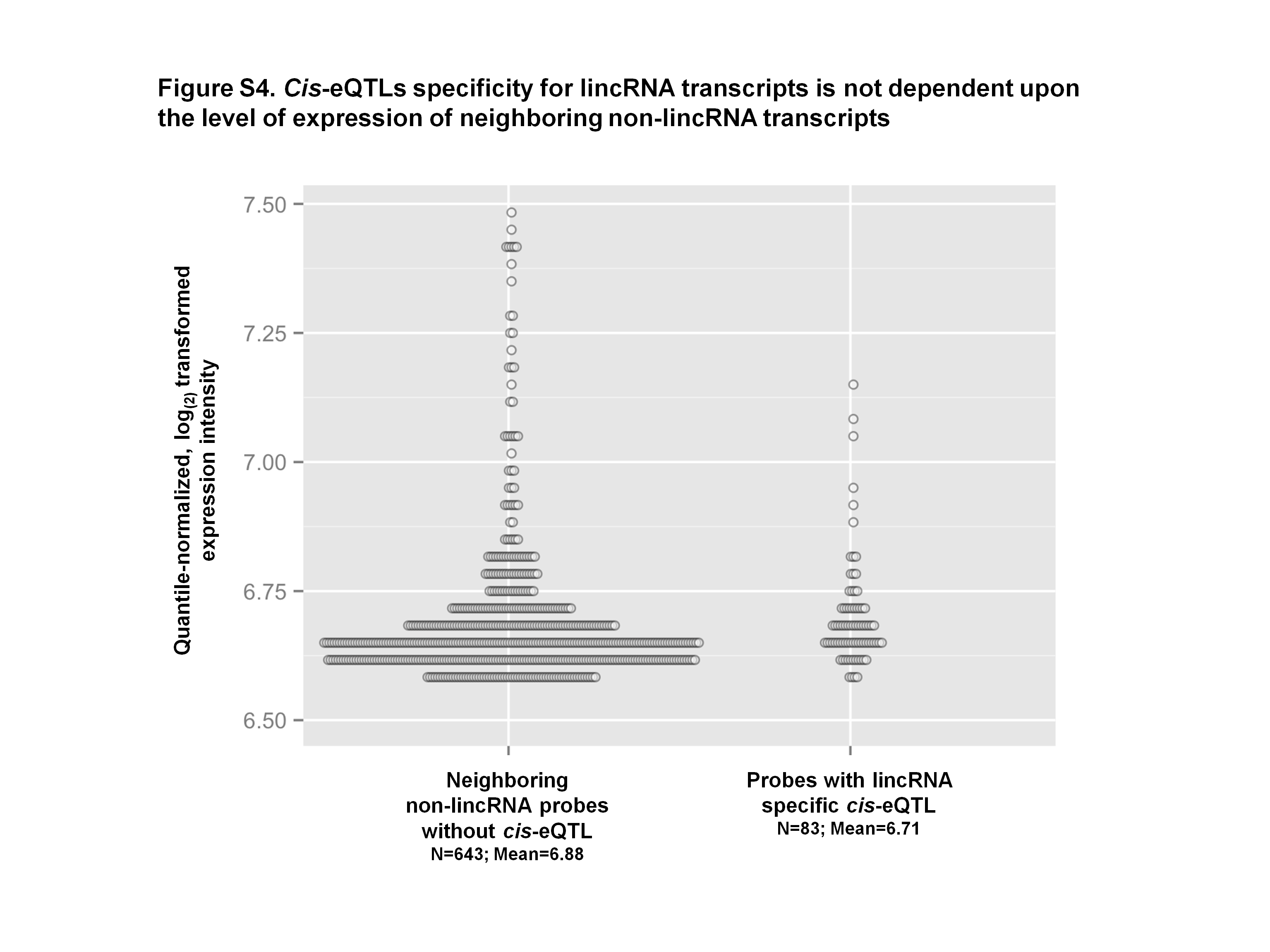

Supplement: Figure S4 — LincRNA cis-eQTL SNPs mostly affect lincRNA transcripts alone. Quantile-normalized average expression intensity of cis-eQTL lincRNAs and their neighboring protein coding genes without cis-eQTL. (TIF) [file pgen.1003201.s004.tif]

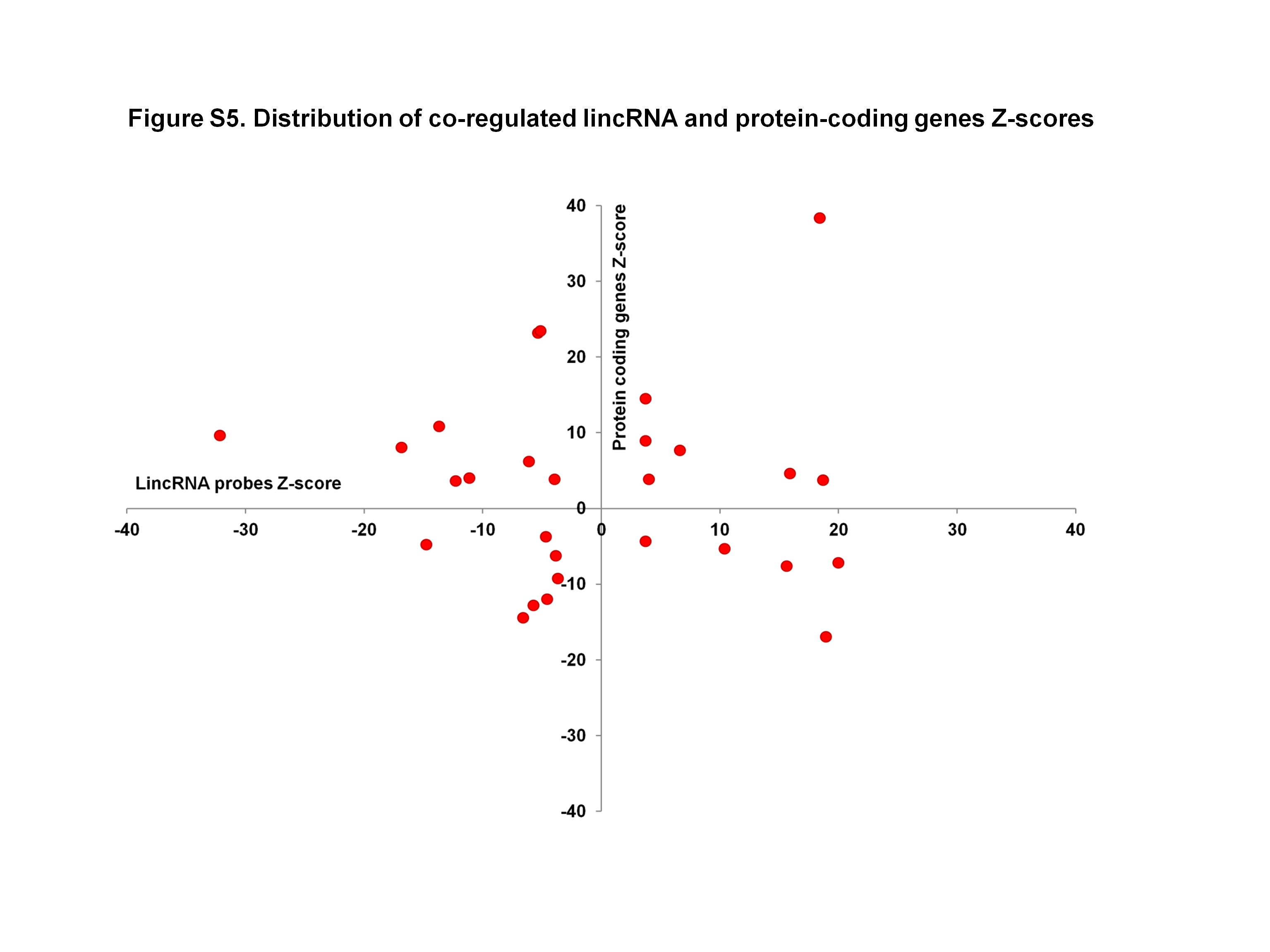

Supplement: Figure S5 — Distribution of Z-scores of co-regulated lincRNA and protein-coding genes. We compared the z-scores (association strength) of each significantly associated probe-SNP pair for the 29 cis-eQTLs that affect both lincRNAs and protein-coding genes. (TIF) [file pgen.1003201.s005.tif]

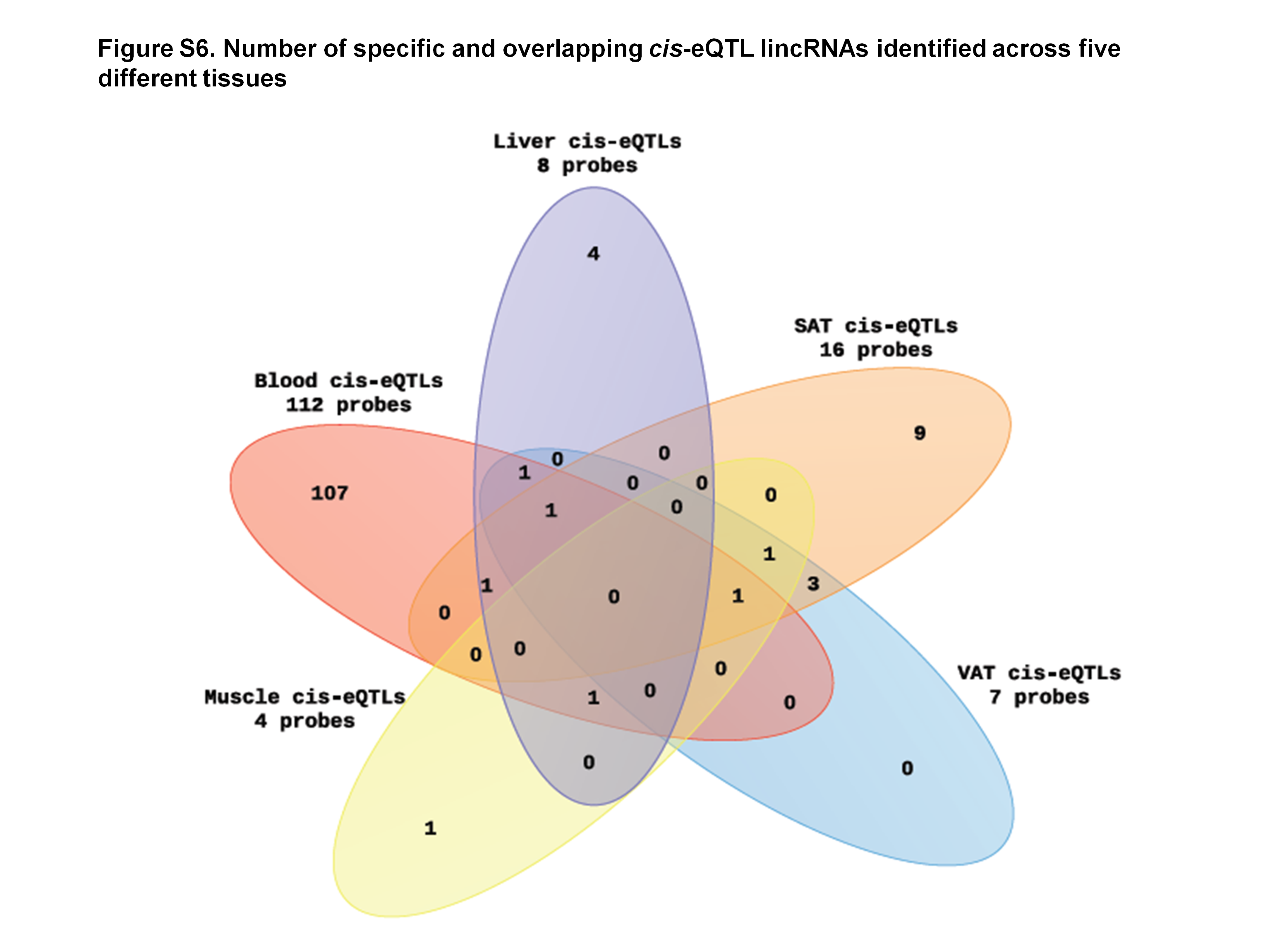

Supplement: Figure S6 — Number of specific and overlapping cis-eQTL lincRNAs identified across five different tissues. (TIF) [file pgen.1003201.s006.tif]

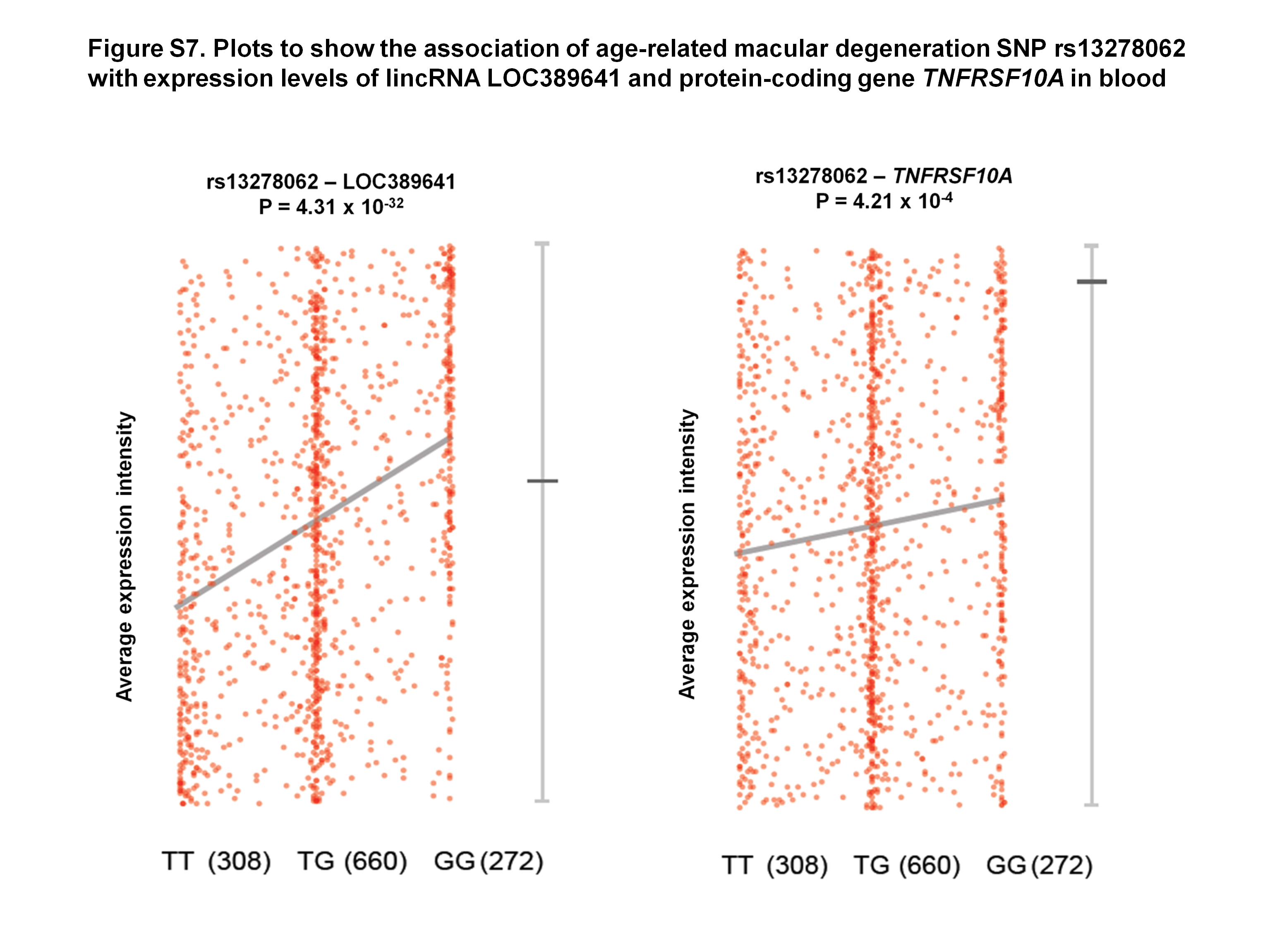

Supplement: Figure S7 — Plots to show the association of age-related macular degeneration SNP rs13278062 with expression levels of lincRNA LOC389641 and protein-coding gene TNFRSF10A in blood (N = 1,249). The x-axis shows the number of samples according to the genotypes at rs13278062 and the y-axis is the average expression intensity of probes. (TIF) [file pgen.1003201.s007.tif]

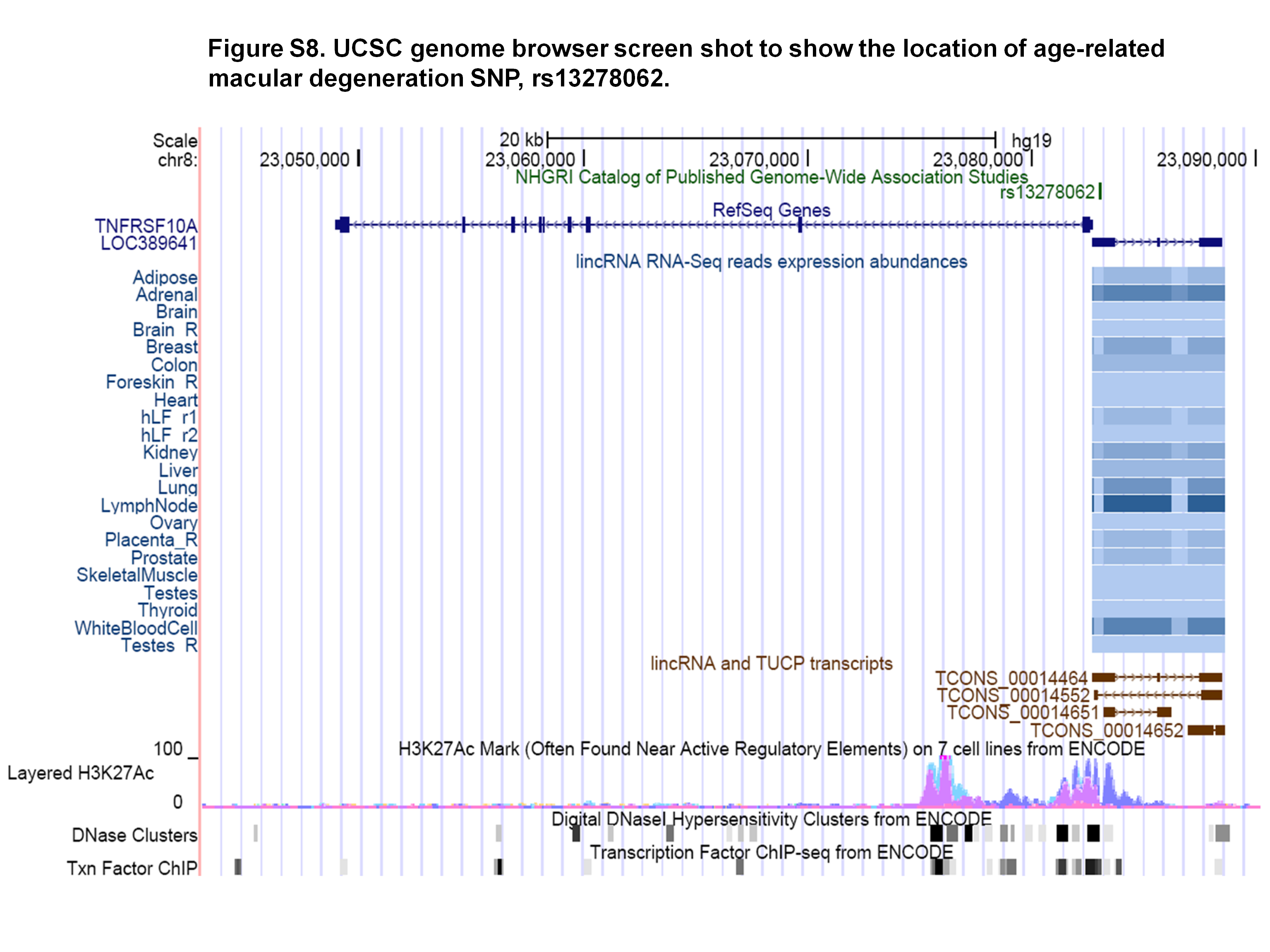

Supplement: Figure S8 — UCSC genome browser screen shot (http://genome.ucsc.edu) to show the location of age-related macular degeneration SNP, rs13278062. The x-axis is the chromosome location in the hg19 build and indicates the location of transcripts and regulatory elements identified by ENCODE on chromosome 8. (TIF) [file pgen.1003201.s008.tif]

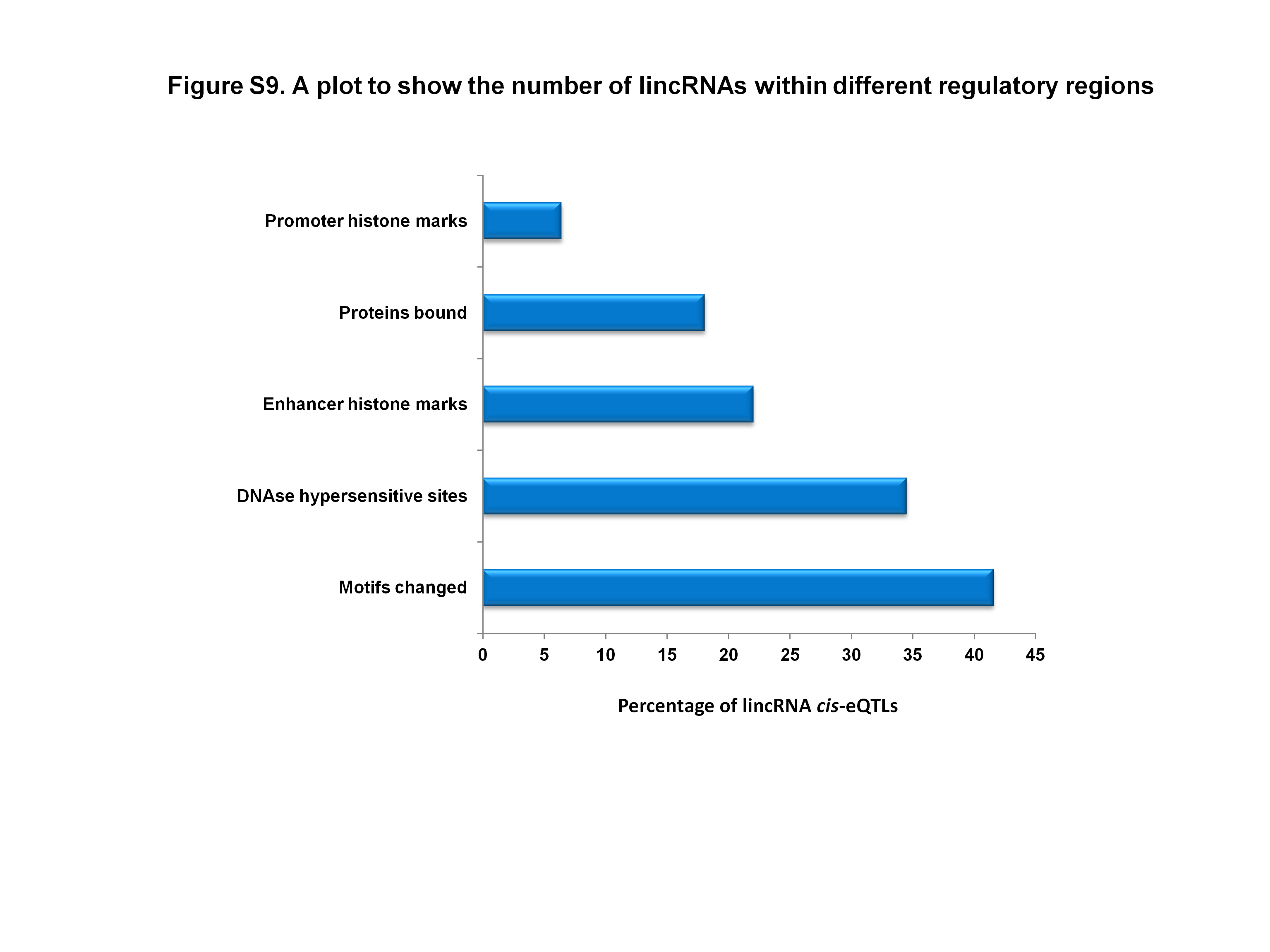

Supplement: Figure S9 — A plot to show the number of lincRNA cis-eQTLs on the y-axis within different regulatory regions on the x-axis. (TIF) [file pgen.1003201.s009.tif]

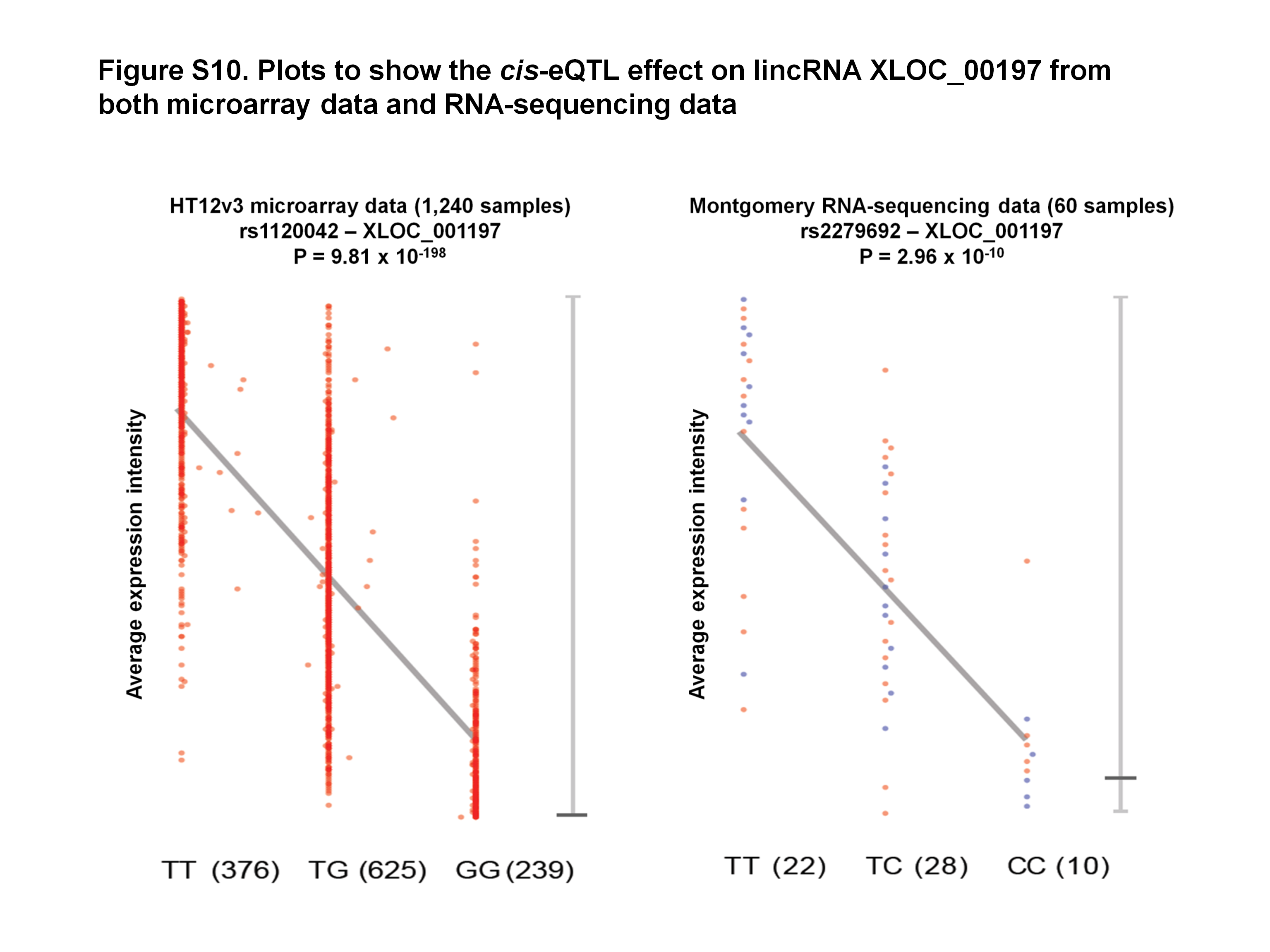

Supplement: Figure S10 — Plots to show the cis-eQTL effect on lincRNA XLOC_00197 from both microarray data (Groningen HT12v3; N = 1,240) and RNA-sequencing data (Montgomery et al; N = 60). The x-axis shows the number of samples according to the genotypes at rs1120042 and rs2279692 (LD between these two SNPs, R2 = 0.96) in microarray data and RNA-sequencing data, respectively. (TIF) [file pgen.1003201.s010.tif]
